# EXAMINING THE NATURE AND ROLE OF FAMILY SYSTEM INTERACTIONS AND ELDER FAMILY FINANCIAL EXPLOITATION

**DOI:** 10.1093/geroni/igz038.1408

**Published:** 2019-11-08

**Authors:** Marlene Stum

**Affiliations:** University of Minnesota, St. Paul, Minnesota, United States

## Abstract

Guided by Bronfenbrenner’s Bioecological Theory, this paper contributes insight into family processes associated with elder family financial exploitation (EFFE). Processes involve “everyday” reciprocal interactions in the family microsystem between and among elder victims, perpetrator family members (i.e. adult children), and involved non-perpetrator family members (i.e. adult children, in-laws). Qualitative data from a purposeful sample of 28 non-perpetrator/non-victim family members who had experienced EFFE were thematically coded and analyzed. The findings suggest eight intergenerational family processes are relevant for understanding EFFE: a) parent/child resource exchange patterns, b) negotiating a “fair” use of resources, c) quality of parent/child and sibling relationships, d) family of origin functioning and dynamics (e.g. trust, respect, closeness), e) communication patterns, f) alliances and taking sides, g) role negotiation, and h) physical interactions. Consistent definitions and quality measures relevant for parent/adult child relationships over the life course and in later life developmental stages are needed next steps.

